# Prednisone and Deflazacort in Duchenne Muscular Dystrophy: Do They Play a Different Role in Child Behavior and Perceived Quality of Life?

**DOI:** 10.1371/currents.md.7628d9c014bfa29f821a5cd19723bbaa

**Published:** 2016-06-17

**Authors:** Susan Sienko, Cathleen Buckon, Eileen Fowler, Anita Bagley, Loretta Staudt, Mitell Sison-Williamson, Kathy Zebracki, Craig M. McDonald, Michael Sussman

**Affiliations:** Clinical Research, Shriners Hospitals for Children, Portland, Oregon, USA; Clinical Research, Shriners Hospitals for Children, Portland, Oregon, USA; Orthopaedic Surgery, University of California, Los Angeles, California, USA; Motion Analysis Laboratory, Shriners Hospitals for Children Northern California, Sacramento, California, USA; Orthopaedic Surgery, University of California, Los Angeles, California, USA; Center for HealthCare Quality Licensing & Certification Division, California Department of Public Health, Sacramento, California, USA; Shriners Hospitals for Children, Northwestern University Feinberg School of Medicine, Chicago, Illinois, USA; Department of Physical Medicine and Rehabilitation, University of California, Davis, Sacramento, California, USA; Shriners Hospitals for Children, Portland, Oregon, USA

## Abstract

The aim of this study was to determine whether prednisone and deflazacort play a different role in child behavior and perceived health related psychosocial quality of life in ambulant boys with Duchenne Muscular Dystrophy. As part of a prospective natural-history study, parents of sixty-seven ambulant boys with DMD (27 taking prednisone, 15 taking deflazacort, 25 were steroid naïve) completed the Child Behavior Checklist (CBCL) for assessment of behavioral, emotional and social problems and both parents and boys with DMD completed the PedsQL™4.0 generic core scale short form. Boys with DMD had higher rates of general behavioral problems than age-matched peers. No significant differences were found among the groups for any of the CBCL syndrome scales raw scores, including internalizing and externalizing behaviors; however, on average boys taking deflazacort demonstrated more withdrawn behaviors than those taking prednisone, while on average the boys taking prednisone demonstrated more aggressive behaviors than boys taking deflazacort. Age, internalizing and externalizing behaviors accounted for 39 and 48% of the variance in psychosocial quality of life for both parents and boys with DMD, respectively. Overall, the use of steroids was not associated with more behavioral problems in boys with DMD. As behavior played a significant role in psychosocial quality of life, comprehensive assessment and treatment of behavioral problems is crucial in this population.

## Introduction

Duchenne muscular dystrophy (DMD) is an X-linked recessive disease of muscle that affects 1 in 6000 live males.^1^ DMD is caused by complete absence of the dystrophin protein in skeletal muscle, myocardium, and brain^2^ and is characterized by a progressive loss of functional muscle mass leading to deterioration in motor milestones with age. Currently, corticosteroids, such as prednisone and prednisolone, are the only US Food and Drug Administration (FDA) approved medication available to stabilize muscle strength, extend ambulation and standing ability, and minimize the incidence of spinal deformity in individuals with DMD. Deflazacort, a similar corticosteroid is currently in clinical trials; however is not approved for use by the FDA. While the benefits of deflazacort are similar to that of prednisone, it is often prescribed because of a perceived reduction in side effects such as weight gain^3^ and undesired behaviors such as aggression.^4^ The decision to initiate the use of corticosteroids depends on functional state, age, and pre-existing risk factors for adverse side-effects;^4^ however, the precise timing of steroid initiation is typically an individual family decision with physician guidance.

There is emerging evidence indicating that the absence of dystrophin results in a disordered architecture of the central nervous system, which affects the brain.^5^ As a result, boys with DMD have an increased risk for behavioral problems, specifically attention deficit hyperactivity disorder (ADHD), autism spectrum disorder, obsessive-compulsive disorder, depression, anxiety, and social difficulties.^6^ In addition, boys with DMD are reported to have difficulties in social functioning that might be due to biologically based deficits in specific social cognitive skills such as social reciprocity, social judgment, and affective discrimination.^4^ These emotional and behavioral problems may lead to social isolation and withdrawal,^4^ with many boys with DMD, regardless of steroid use, becoming more withdrawn, depressed and isolated as they get older.^7^

Anecdotally, parents report an increase in negative behaviors, specifically mood swings, difficult behavior and aggression, with the initiation of corticosteroids; however, research evaluating the impact of corticosteroids on behavior and emotional difficulties remains equivocal.^3^^,^ 6 ^,^ 8 A recent study by Caspers Conway et al reported that corticosteroid and mobility device use were associated with behavior problems.^9^ Studies by both Hinton et al. and Hendriksen et al. have both found that steroids were not associated with the behavioral outcomes of the children in their studies.^5^^,^ 10 Hendriksen et al. reported that there was no significant difference in overall social adjustment between males with DMD taking corticosteroids and those who were not and that there was no indication that steroids played a major role in negatively modifying psychological adjustment.^5^ Similarly, Hinton et al. found that steroid use was not the primary factor contributing to an increase in social problems, reported by the parents.^10^ Despite the focus on the deterioration in motor capacity associated with DMD, parents report that emotional and behavioral problems are a significant issue, which can also negatively affect their son’s quality of life.^11^^,^ 12

To gain further insight into the impact that corticosteroids may have on behavioral and emotional problems and health related quality of life (HRQOL) in ambulant boys with DMD, the objective of this study was to determine whether prednisone and deflazacort play a different role in child behavior and perceived psychosocial quality of life.

## Methods

Participants

Participants included boys with DMD, between the ages of 4 to 15 years who were ambulatory and were part of a multicenter (Shriners Hospitals for Children-Portland, Shriners Hospitals for Children-Northern California or The University of California-Los Angeles) natural history study focusing on the biomechanics of gait in boys with DMD. Eighty-five boys with DMD participated in the longitudinal study; however, at the time of implementation of a measure assessing emotional, behavioral and social functioning into the protocol only 67 boys remained ambulatory, were still participating in the study and completed this measure. In this paper, we investigate the initial behavioral assessments and health related quality of life using a cross-sectional design. Confirmation of Duchenne muscular dystrophy was based on typical clinical presentation of Duchenne muscular dystrophy and one or more of the following: documentation of disease-causing mutation in the dystrophin gene, elevated serum creating kinase levels, and a family history of an affected relative with either a disease-causing mutation in the dystrophic gene and/or complete dystrophin deficiency as shown by immunostaining on muscle biopsy. The Institutional Review Boards from all participating centers approved this study. All parents/guardians provided informed consent and boys provided assent when appropriate.

Questionnaires

As part of the longitudinal study, parents completed the Child Behavior Checklist (CBCL) 1½-5 or 6-18 years.^13^ The CBCL is a 118-item questionnaire assessing behavioral, emotional, and social problems rated on a 3-point scale ranging from 0 (Not True) to 2 (Very True or Often True).^13^ In this study, we only used the syndrome scales of the CBCL. Syndromes are sets of concurrent problems that tend to co-occur together. Syndrome scales include anxious/depressed, withdrawn/depressed, somatic complaints, social problems, thought problems, attention problems, rule-breaking behavior and aggressive behavior. Syndrome scales are categorized into internalizing and externalizing behaviors. Internalizing behaviors are problems that are primarily within the individual and include anxious/depressed, withdrawn/depressed and somatic complaints, while externalizing behaviors are problems that mainly involve conflict with other people and their expectations for the child and include rule-breaking and aggressive behavior subscales.^14^ Higher scores on the CBCL indicate more problems. In addition to the completion of the CBCL, parents and boys with DMD completed the PedsQL™ 4.0 generic core scale short form (SF-15).^15^^,^ 16 ^,^ 17 For boys between the ages of 5 and 7 and in situations where the child was unable to read or complete the questionnaire independently because of cognitive impairment, the PedsQL™ was interview-administered by one of the researchers. The PedsQL asks “how much of a problem has your child had with…,” with a score of 0=never and a score of 4=almost always. The PedsQL is reversed scored so that a score of 100 means that the parent/child did not report any problems in this area.

Statistical Analysis

Boys were divided into three groups according to their parent-initiated use of corticosteroids: prednisone, deflazacort, and steroid naïve (had not started taking steroids at the time of the initial CBCL assessment). While power was not determined for the behavior assessment, power calculations for the primary outcomes (gait and energy cost) determined that a sample size of 68 subjects was needed to obtain 80% power to detect a correlation of 0.3 or greater between gait, energy and functional variables at p< 0.05; however, a sample size of 85 was recruited to account for potential subject drop out over the study period. To examine whether the behavior profiles for the syndrome scales differed among the three groups one-way ANOVAs of the raw CBCL scores were used. For the syndrome scales, T scores below 65 are considered within the normal range, while scores between 65 and 69 are considered borderline clinical behaviors and scores greater than 70 are considered clinical behaviors.^14^ Using 65 as the cutoff, boys were classified as having or not having borderline/clinical behaviors. Crosstabs were used to determine whether the proportion of boys falling into the borderline/clinical category differed by parent-selected corticosteroid group.

For the PedsQL, Pearson correlation coefficients and intraclass correlation coefficients (ICC 2,1) were used to determine agreement in quality of life domains between parents and their sons. ICC values were considered poor to fair if ≤.40, moderate if 0.40-0.60, good it 0.61-.80 and excellent if 0.81 to 1.0.^8^ A mean psychosocial health summary score was computed by summing the items from the emotional, social, and school functioning scales and dividing by the number of items.^18^ Due to the small number of subjects and the large number of subscales within the CBCL, only the summary scores from the internalizing and externalizing behaviors were used in the regression analysis. Hierarchical linear regressions were used to determine whether age and internalizing and externalizing behaviors predicted parent proxy and child reported psychosocial health quality of life. In order to account for the influence of age on behaviors, age was entered at step one, followed by internalizing and externalizing behaviors at step 2. Significance was set at p < 0.05.

## Results

Population Characteristics

All 67 boys were ambulant without assistive devices. 27 boys were taking prednisone (mean age 8 years, 5 months), 15 boys were taking deflazacort (mean age 9 years, 8 months), and 25 boys were not taking steroids or had not started taking steroids at the time of the evaluation (mean age 6 years, 5 months). The boys using steroids (prednisone and deflazacort) were significantly older than those who were steroid naïve, p=.01. No differences in age were found between the steroid groups (p=.38).

Behavior of boys with DMD and the influence of steroids

No significant differences were found among the groups for any of the CBCL syndrome scales raw scores, including internalizing and externalizing behaviors (Table 1). Mean T-scores for the syndrome scales (anxious/depressed, withdrawn/depressed, somatic complaints, social problems, thought problems, attention problems, rule-breaking behavior and aggressive behavior) fell within normal limits (<65) for all corticosteroid groups. There were no differences in the percentage of boys with T-scores in the borderline/clinical category within each parent-selected corticosteroid group for any of the behavioral subscales, including internalizing and externalizing. While not statistically significantly different, greater than 30% of boys on prednisone had T-scores in the borderline/clinical range for externalizing behaviors (rule breaking and aggressive behaviors), while greater than 30% of boys on deflazacort had T-scores in the borderline/clinical range on the withdrawn/depression syndrome subscale (Figure 1).

**Table 1: CBCL Results Raw and T scores for the total group and by parent-selected corticosteroid group:** Means (standard deviations)

Variable	Total Group (n=67)		No Steroid (n=25)		Prednisone (n=27)		Deflazacort (n=15)		ANOVA
	T Score	Raw	T Score	Raw	T Score	Raw	T Score	Raw	Significance
Internalizing	53 (11)	8 (6)	52 (12)	8 (6)	55 (10)	8 (5)	53 (10)	8 (6)	p=.96
Externalizing	52 (11)	10 (8)	49 (14)	10 (10)	55 (9)	11 (7)	51 (10)	8 (8)	p=.66
Anxiety/Depressed	55 (7)	10 (8)	55 (7)	3 (3)	55 (7)	4 (4)	55 (6)	3 (3)	p=.88
Withdrawn/Depressed	57 (7)	2 (2)	56 (6)	2 (2)	58 (6)	2 (2)	57 (9)	2 (3)	p=.80
Social Problems	59 (8)	5 (4)	61 (12)	6 (5)	60 (7)	5 (3)	57 (7)	3 (3)	p=.28
Attention Problems	57 (7)	5 (4)	56 (7)	4 (5)	57 (7)	5 (4)	57 (7)	5 (4)	p=.72
Aggressive Behavior	56 (8)	7 (7)	56 (10)	7 (8)	58 (7)	8 (5)	55 (7)	6 (6)	p=.57

**Bordeline/Clinical CBCL Results:**
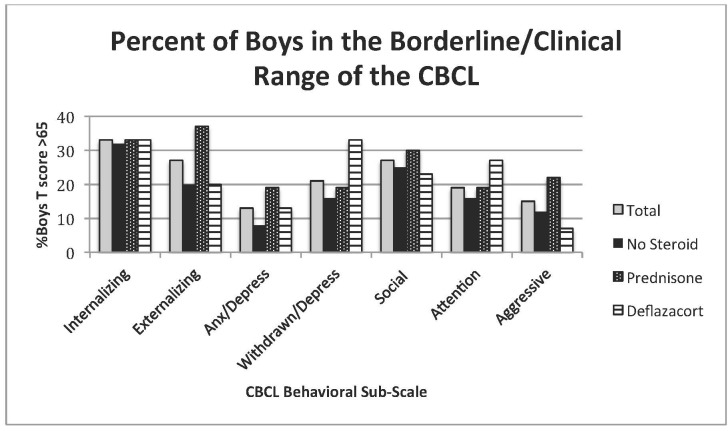
Percent of boys with T scores in the Borderline/Clinical Range (>65) for the total group and by parent-selected steroid group

Agreement in perceived quality of life between boys with DMD and their parents

Agreement on the PedsQL between parent proxy and child self-report is shown in Table 2. Overall, boys with DMD self-reported lower quality of life than parent proxy in all domains, except school functioning. Moderate agreement between the boys and their parents was found for physical functioning (r=.49, ICC=.48) and social functioning (r=.42, ICC=.42) only. The agreement between boys and their parents for psychosocial health, which includes emotional, social and school functioning was considered poor to fair (r=.36, ICC=.36). Agreement between boys and their parents for emotional (r=.25, ICC=.24) and school functioning (r=.32, ICC=.36) were considered poor to fair.

**Table 2: PedsQL Means and agreement indices for parent-son reports of quality of life:** sd=standard deviation, ICC=intraclass correlation coefficient, CI=confidence interval **Correlation is significant at the 0.01 level (2-tailed) * Correlation is significant at the 0.05 level (2-tailed)

PedsQL Item	Parent, Mean (sd)	Child, Mean (sd)	r	ICC (95% CI)
Physical Health	43.2 (27.6)	42.9 (22.5)	.49**	.48 (.20-.69)
Psychosocial Health	66.2 (15.6)	64.2 (15.4)	.36*	.36 (.05-.61)
Emotional Functioning	66.5 (16.7)	63.2 (21.3)	.25	.24 (-.07-.52)
Social Functioning	72.7 (22.8)	67.3 (23.8)	.42**	.42 (.13-.65)
School Functioning	59.7 (20.6)	61.7 (21.3)	.32	.32 (.00-.58)

The influence of behavior problems on psychosocial quality of life

For parent-proxy reported psychosocial quality of life, the variables of age and internalizing and externalizing behaviors accounted for 39% of the variance in psychosocial quality of life F(3,63)=12.79, p<.001. At step 1 age accounted for a significant amount of variance (R^2^=.08), while the addition of internalizing and externalizing behaviors at step 2 resulted in a significant increase in the amount of explained variance (R^2^=.39, F_inc_ (2, 60) =15.09, p<.001) Psychosocial quality of life was negatively associated with age (b=-.25, p=.018) and internalizing behaviors (b=-.36, p=.007). A similar pattern was also seen for psychosocial quality of life as self-reported by the boys with DMD. Age and internalizing and externalizing behaviors accounted for 48.5% of the variance in psychosocial quality of life F=(3, 33)=10.35, p<.001. At step 1, age accounted for a significant amount of variance (R^2^=.15), while the addition of internalizing and externalizing behaviors at step 2 resulted in a significant increase in the amount of explained variance (R^2^=.34, F_inc_ (2,33)=10.87, p<.001). In contrast to the parents, self-reported psychosocial quality of life was positively associated with age (b=.31, p=.02). Similar to the parents, age was negatively associated with internalizing behaviors (b=-.45, p=.01).

## Discussion

Social and behavioral issues have been reported in boys with DMD, with parents reporting that these problems are exacerbated with the use of steroids. Similar to the findings reported in the literature,^10^^,^ 19 boys with DMD in this study had higher rates of behavioral problems than age-matched peers. Despite slightly higher rates of behavioral problems for the entire cohort, no statistically significant differences were found between steroid usage groups. These findings are similar to those of Hinton et al^10^ and Hendriksen et al^5^ who found that steroid usage was not significantly associated with behavioral outcomes. While not statistically significant, there were differences in the patterns of behavior seen among the three groups. Overall, the boys not taking steroids were less likely to be in the borderline/clinical range for any of the behavioral subscales. A greater percentage of boys taking prednisone had increased externalizing behaviors in the borderline/clinical range, which include rule-breaking and aggressive behaviors, while boys taking deflazacort demonstrated greater internalizing behavior problems associated with withdrawal and depression. It has been reported that boys with DMD who were using steroids showed less withdrawal than boys who were not using steroids;^5^^,^ 8 however, this is not consistent with the findings of this study. Solden et al^20^ reported that boys with DMD were more likely to have problems with anxiety-withdrawal than with aggression. In our cohort the behavioral tendencies, although not significant, differed by steroid use. The behavioral differences between the boys taking steroids and those who are not may be a function of age rather than medication, as the boys not on steroids were two to three years younger than boys taking steroids. While problems with behavior regulation, specifically oppositional-defiant and aggressive behaviors^21^ have been reported in boys with DMD, a greater percentage of boys with DMD demonstrated problems with internalizing behaviors rather than externalizing behaviors, which may be associated with having a chronic degenerative illness.

While self-report is considered the gold standard for measuring quality of life, parents are often asked to provide an assessment of their child’s quality of life. Proxy-reporting is problematic due to the inconsistencies in the level of agreement between the parent and child.^22^ Similar to the findings in the literature,^8^^,^ 23 ^,^ 24 ^,^ 25 boys with DMD and their parents showed poor to moderate agreement on perceived quality of life.^24^ Observable symptoms, such as physical functioning are highly consistent between the parent and child; however, internal domains such as school and emotional functioning yield poorer agreement.^8^ In previous studies, parents of boys with DMD tend to underestimate the health-related quality of life perceived by their sons.^22^^,^ 25 In contrast to Bray et al.,^22^ the boys with DMD in this study reported poorer quality of life than their parents for emotional and social functioning. The lack of agreement between the boys and their parents reflects the need for self-report questionnaires whenever possible in order to obtain the appropriate reference point.

Families of boys with DMD report that the quality of life and the mental health of their son are of major concern for them.^26^ Overall, both boys and their parents reported lower quality of life across all quality of life domains as compared to same-aged peers, similar to the results reported by Bendixen et al.^11^ In contrast to the literature, parents reported a decrease in psychosocial quality of life with increasing age. According to parental proxy data, decreased psychosocial quality of life and increased internalizing behaviors occurred as a function of age. This decrease in psychosocial quality of life may be related to reports in the literature that as boys with DMD get older they tend to become more withdrawn, depressed, and isolated.^27^ Additionally, as boys with DMD age, they may be physically unable to act out and demonstrate externalizing behaviors such as hitting. Similarly, increased internalizing behaviors such as anxiety, withdrawal and depressive symptoms were associated with decreased quality of life per child report. As emotional factors such as anxiety and depression have been found to contribute to peer problems for boys with DMD,^5^ this may further affect psychosocial quality of life. Despite the decreased psychosocial quality of life with increasing age reported by the parents, reports from boys with DMD were more consistent with the literature^5^^,^ 8 reporting an increase in psychosocial quality of life with increasing age. It is unclear from this study what factors influence the boys’ perceptions of improved psychosocial quality of life and how these differ from their parents.

## Limitations

The purpose of this study was to examine whether prednisone and deflazacort play a different role in parental reported behavior problems and psychosocial quality of life in boys with DMD, as measured by the CBCL and the PedsQL. The results from this study are based on both parent and child report and may represent some bias. In this study the assessment of emotional and behavior issues by the CBCL was completed by the caregiver only and did not incorporate input from the teacher and or a psychologist, which may bias the results. In situations where the child was too young to read or cognitive ability prevented them from completing the assessment independently, assistance with reading was provided by the researcher. Although the child answered the PedsQL independently by pointing to the appropriate face response, answers may be influenced by researcher intervention. While the subjects from this study were recruited from three different geographical centers, this sample may not be representative of boys with DMD as a group, due to the small number of subjects. Additionally, age may influence the commencement of behavioral problems; however, the sample size in this study prevented the stratification of results by both age and steroid regime. Additionally, the CBCL may not be the optimal tool to use with boys with DMD as it may be overly sensitive to living with a chronic condition and physical disabilities and may over-represent psychosocial maladjustment.^5^^,^ 28 Although steroid grouping was based on parent-selected choice, other medication factors such as compliance, dose, and duration of use were not controlled for in this study. Recently, the role of dystrophin and the disruption of GABA_A_ receptors in the brain have received increasing attention with respect to their impact on behavioral and cognitive dysfunction.^29^ The higher rates of behavioral problems and the variability of the entire cohort may be a result of the lack of dystrophin. The interaction between the corticosteroids and the GABA_A_ receptors is another factor that may affect the efficacy of these medications.^29^ In addition, the results of the behavioral assessment may be influenced by the use of concomitant medications. In contrast to the findings of Caspers Conway et al, who reported that 25% received neuropsychiatric medication and 27% received both counseling and medication^9^ to address behavioral issues, only 3 boys in our study were taking medication to address behavior issues. One boy in the steroid naïve group was taking clonazepam for panic attacks and methylphenidate for ADHD, which may impact the parent perceptions of behavioral problems. Similarly, two boys in the deflazacort group were taking fluoxetine, which may affect scores within the internalizing behavior domains.

## Summary

Similar to the findings from Caspers Conway et al.^9^ , our study found that boys with DMD exhibit more behavioral problems than their peers and have a greater number of internalizing problems as compared to externalizing behaviors. As more information about the role of dystrophin in the brain becomes available,^29^^,^ 30 the etiology of neurobehavioral problems in boys with DMD may be better understood. Overall, the use of steroids was not associated with more behavioral problems in boys with DMD; however, slight differences in the types of undesired behaviors were evident between boys taking deflazacort and those taking prednisone; however, the longitudinal data from this natural history study may provide insight changes over time. As behavior played a significant role in psychosocial quality of life, comprehensive assessment and treatment of behavioral problems is crucial in this population.
